# Malondialdehyde Level and Tissue Apoptosis Count as an Early-Detection Marker of Oral Potentially Malignant Disorders

**DOI:** 10.1055/s-0042-1743154

**Published:** 2022-04-18

**Authors:** Amalia Yosi Firdausa, Sally Salsabila Ahimsa, Rafdan Affan Ahmada, Natasya Fauzia Sukmawati, Diah Savitri Ernawati, Adiastuti Endah Parmadiati, Bagus Soebadi, Desiana Radithia, Saka Winias, Fatma Yasmin Mahdani, Riyan Iman Marsetyo, Rosnah Binti Zain, Nurina Febriyanti Ayuningtyas

**Affiliations:** 1 Faculty of Dental Medicine, Universitas Airlangga, Surabaya, Indonesia; 2 Department of Oral Medicine, Faculty of Dental Medicine, Universitas Airlangga, Surabaya-Indonesia; 3 Faculty of Dentistry, MAHSA University, Bandar Saujana Putra, Jenjarom Selangor, Malaysia; 4 Oral Cancer Research and Coordinating Centre, Faculty of Dentistry, University of Malaya, Malaya, Malaysia

**Keywords:** tobacco addiction, apoptosis index, malondialdehyde, oral epithelial dysplasia, oral potentially malignant disorder, cancer

## Abstract

**Objectives** The malondialdehyde (MDA) level and TA count represent the progression of oral potentially malignant disorders (OPMD) to malignancy and thus may be used as an indicator of oral epithelial dysplasia (OED). This study aimed to determine the MDA level and tissue apoptosis (TA) count in oropharyngeal tissue of Wistar rats exposed to sidestream cigarette smoke.

**Materials and Methods** Wistar rats were divided into three groups: T4 group (4-week cigarette smoke exposure), T8 group (8-week cigarette smoke exposure), and control group, which was not exposed to cigarette smoke. The oropharyngeal tissue of the rats from each group was examined histopathologically to count the number of apoptotic cells, and then the blood serum was made to measure the MDA level.

**Statistical Analysis** Bonferroni test was performed to see the differences in each group for MDA level. While the data from tissue apoptosis were analyzed using Mann-Whitney U test for the significance. All data were considered significant if *p* < 0.05.

**Results** The MDA level and TA count increased as the duration of cigarette smoke exposure increased. In the T8 group, the MDA level and TA count were significantly higher compared with the T4 and control groups with a *p* -value < 0.05.

**Conclusions** Exposure to sidestream cigarette smoke increased the TA count and MDA level in the oropharyngeal tissue of Wistar rats. The TA count and MDA level may be used as markers of OPMD.

## Introduction

Oral cancer is the sixth most deadly cancer in the world with an estimated 300,400 cases each year, which accounts for 145,400 deaths each year. The etiology of oral cancer includes smoking and alcohol consumption. 1 There are more than 5 million deaths of cancer patients in the world caused by cigarette smoke every year. 2 Cigarette smoke is divided into mainstream smoke and sidestream smoke. 3 A causal relationship between mainstream smoke and cancer has been established. Lee et al showed that 20 to 30% of cigarette-related diseases, including cancer, are caused by sidestream smoke. 2 Sidestream cigarette smoke contains higher concentrations of toxic compounds and carcinogens than mainstream smoke. This is due to incomplete combustion that results in the presence of more complex compounds in sidestream smoke. 4

Under the normal physiologic condition, cells can regulate the balance between reactive oxygen species (ROS) formation and its disposal, but exposure to cigarette smoke can increase ROS level and trigger loss of homeostasis. Uncontrolled increases of ROS in the body are called oxidative stress conditions. 5 In the early stages of the precancerous and neoplastic phase, oxidative stress can induce the cancer initiation phase, which then indirectly contributes to other stages of cancer, namely promotion and cancer progeny. 6 Malondialdehyde (MDA) is the result of ROS damage to fat. Fat is very easily affected by ROS because fat has a sensitive double bond, so ROS makes lipid peroxidation that leads to malignancy. MDA is a product of lipid peroxidation that has high cytotoxicity and has tumor promoter properties and carcinogenic agents in the blood. 7

In addition, chronic cigarette smoke exposure can induce carcinogenesis through apoptotic resistance. 8 Apoptosis is programmed cell death, which is a part of the normal physiological process to destroy cells that are not needed. 9 Apoptosis is characterized by changes in cell morphology, namely chromatin fragmentation accompanied by shrinkage and destroyed cell nuclei, which are called apoptotic bodies. 10 Apoptosis requires a protease called caspase whose activation can be achieved by the presence of signals from outside the cell (extrinsic apoptosis pathway) and signals internally such as DNA damage (intrinsic apoptosis pathway). 11 12 Dysregulation of the apoptotic pathway often contributes to cancer development and resistance to cancer therapy. 13

Cancer in the oral cavity can be preceded by oral mucosal changes that have the potential for malignant transformation called oral potentially malignant disorders (OPMD). OPMD in the oral cavity includes the presence of lesions such as leukoplakia, erythroplakia, erythroleukoplakia, lichen planus, and oral submucous fibrosis. 14

Early diagnosis of OPMD can increase a patient's chances of recovery and significantly reduce the deformity arising from advanced cancer and/or the outcome of treatment. Identification and quantifying cell apoptosis histologically is one of the ways to detect premalignancy. 15 MDA level in the blood are measured to determine the body's ability to resist oxidative stress, which is also expected to be a marker of premalignancy by exposure to cigarette smoke. 16 This study aimed to determine the MDA level changes in the blood serum and TA count from oropharyngeal tissues of Wistar rats exposed to sidestream cigarette smoke. Measuring these parameters to indicate premalignancy may lead to better prognosis for the patient.

## Materials and Methods

This study was an *in vivo* laboratory experiment on Wistar rats ( *Rattus norvegicus* ) and using a randomized posttest-only control group design. All experimental animals were treated by following the regulations on the Animal Care and Use Committee of Universitas Airlangga. The study protocol was approved by the Health Research Ethical Clearance Commission, Faculty of Dental Medicine, Universitas Airlangga, with the registration number 117/HRECC.FODM/VII/2018.

### Animal

This study used Wistar rats ( *Rattus norvegicus* ) with the distribution shown in Table 1 .

**Table 1:** Animal distribution

Qualifications of Wistar rats ( *Rattus norvegicus* )	
Age	3 mo old
Weight	170 g (±10%)
Health status	Clear-eyed, shiny fur, agile movements, no weight loss for 1 wk or during the adaptation period
Number of rats	27 individuals
Grouping	1. Control group (not exposed to sidestream cigarette smoke) 2. T4 group (exposed to sidestream cigarette smoke for 4 wk) 3. T8 group (exposed to sidestream cigarette smoke for 8 wk)

### Cigarette Smoke Exposure

The exposure of sidestream cigarette smoke to Wistar rats was performed using a smoking pump that connects cigarette smoke from the combustion pipe into a tube containing Wistar rats. These smoking pump toolboxes consist of 10 boxes of the same size 15 × 7 × 7 cm each with a capacity of 1 rat per box. The rats were exposed to cigarette smoke by 20 clove cigarettes per exposure (1 cigarette contains 2.1 mg of nicotine) per day, with total smoke exposure received by each group in 1 day amounting to 20 cigarettes. The exposure was performed for 4 and 8 weeks for the T4 and T8 groups, respectively. 17

### MDA Serum Preparation

Preparation of MDA serum started with 27 Wistar rats that were given inhalation of ethyl ether together with administration of ketamine solution and 2-hydrochloride (2.6 xylidine)-5,6-dihydro-4H-1,3-thiazine, at a dose of 100 and 10 mg/kg intramuscularly on the inside of the left thigh until the rats have been anesthetized. Then, 3 mL of blood was withdrawn from the left ventricle of the rat's heart and put into a plain tube. After that, the rat was sacrificed. Then, 3 mL of rat blood was centrifuged in a centrifugation device at 4,000 rpm for 10 minutes to obtain the blood serum. The serum was taken as much as 0.5 mL and 4.5 mL of PBS was added, and then homogenized. After that, 1 mL of the solution was removed from the mixture. Next, 1 mL of 15% trichloroacetic acid (TCA) was added to the solution. After that, 0.37% of 1-mL thiobarbituric acid (TBA) in 0.25-N HCl was added and then homogenized. After homogenization, the mixture was heated in 80°C water bath for 15 minutes. Then, it was cooled at room temperature for 60 minutes. The mixture was then centrifuged at 3,000 rpm for 15 minutes. The results were measured using MDA samples on a spectrophotometer at 532 nm and the MDA level was calculated using the regression line equation from the standard curve of the MDA solution.

### Tissue Preparation for Apoptotic Cell Count

Apoptotic cells in oropharyngeal tissue preparations were identified and quantified by a histopathologist from sections stained with hematoxylin and eosin (HE) under a light microscope with 400X magnifications and eight visual fields were selected without artifacts. The criteria for the identification of apoptotic cells are the following:

1. presence of chromatin fragmentations in the nuclei.
2. cell membrane shrinkage.
3. thick dark eosinophilic cytoplasm.
4. solid pyknotic nuclei with a round, oval, or irregular shape.

### Statistical Analysis

Statistical Package for the Social Science Software (SPSS) was used for statistical analysis. For the MDA level, the results of the Shapiro–Wilk test showed the data are not normally distributed ( *p* ≤ 0.05). The results of the Levene test show that the data are not homogeneous ( *p* < 0.05). Because the data were not normally distributed and nonhomogeneous, additional tests were needed, namely cooperative testing using the Kruskal–Wallis test. Then, the Bonferroni test was performed to see the differences in each group. The data from the tissue apoptosis results were analyzed statistically by nonparametric tests. The significance test used was the Kruskal–Wallis test, followed by the Mann–Whitney *U* test.

## Results

### MDA Level

The MDA levels calculated from the blood serum of Wistar rats are listed in Table 2 . The results indicated a significant increase ( *p* = 0.000) in the mean value of MDA, which is directly proportional to the length of exposure to cigarette smoke, with the MDA level in the T8 group (7.35 ± 1.43) higher than those in the T4 group (5.85 ± 1.23) and the control group (1.50 ± 0.50; *p* = 0.029 and 0.000, respectively).

**Table 2:** Mean value of malondialdehyde (MDA) level and tissue apoptosis on control, T4, and T8 groups

Marker	Control	T4 group	T8 group	*p* -Value
MDA level (nmol/ml)	1.50 ± 0.50 a,b	5.85 ± 1.23 a,c	7.35 ± 1.43 b,c	0.000 a,b 0.029 c
Tissue apoptosis	1.00 ± 0.00 d,e	7.00 ± 1.22 d,f	9.70 ± 1.09 e,f	0.000 d,e 0.001 f

Abbreviations: T4, the 4-week exposure group; T8, the 8-week exposure group.

Note: Data presented as mean ± standard deviation.

a–c Significant differences result using Bonferroni's test, with *p* < 0.05.

d–f Significant differences result using the Mann–Whitney *U* test, with *p* < 0.05.

### Tissue Apoptosis Count

The results of the apoptotic cell count observed from the oropharyngeal tissue are listed in Table 2 . There was a significant increase ( *p* = 0.000) in the number of apoptotic cells, which is directly proportional to the length of exposure to cigarette smoke. Apoptotic cells in the T4 group (7.00 ± 1.22) were higher than those in the control group (1.00 ± 0.00) with *p* = 0.000. In the T8 group (9.70 ± 1.09), the number of apoptotic cells was significantly higher than that in the T4 and control groups ( *p* = 0.001 and *p* = 0.000, respectively).

**Fig. 1:**
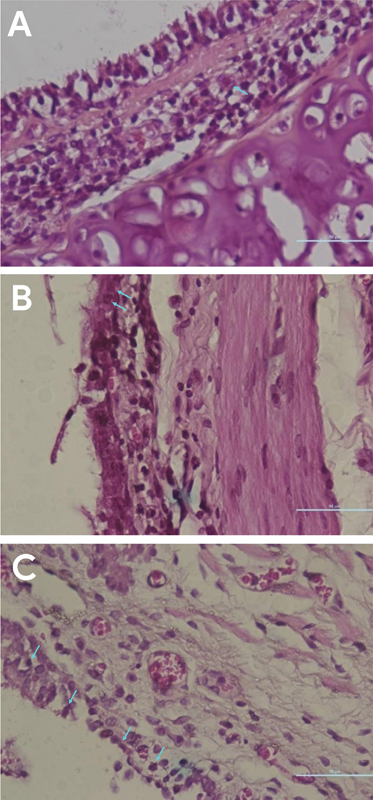
Apoptotic cells in oropharyngeal tissue ( *blue arrow* ) in ( **A** ) the control group sample, ( **B** ) the 4-week exposure group sample, and ( **C** ) the 8 week exposure group sample.

The picture of cells undergoing apoptosis in histologic preparation of experimental animals can be seen in Fig. 1 . In tissue stained by HE, apoptotic cells showed morphological changes in the form of chromatin fragmentation, accompanied by cell membrane shrinkage, thick dark eosinophilic cytoplasm, and solid pyknotic nuclei with a round, oval, and irregular shape. More apoptotic cell images were found in the T8 group sample than in the T4 group sample, but only one cell was found showing a picture of apoptosis in the control group sample.

## Discussion

One of the biomarkers of cancer and OPMDs is MDA, which when increased may indicate the increase in ROS. 18 Uncontrolled increase of ROS affects fat, resulting in fat peroxidation with MDA as one of the residual products. MDA has high cytotoxicity and has tumor-promoting properties and carcinogenic agents in the blood. 16 MDA indicates DNA damage in the process of fat peroxidation. 19 The increase in MDA concentration occurs in the state of cells that turn into tumors and is seen in the early stage of cancer. 15 18 In addition, the MDA processing technique is easy because MDA reacts to the TBA (thiobarbituric acid) reagent in the form of a color change to a fluorescent red color so that MDA is selected as a biomarker for this study. 20

The increase in MDA concentration in serum or blood plasma always occurs in various types of cancer. 18 Persistent exposure to cigarette smoke will increase the level of ROS and other chemicals in the blood, which are leftover products in the body. The toxic materials from sidestream smoke are known to be more easily absorbed in the blood and lymph glands compared with mainstream smoke. 21 In addition, examination using blood is expected to be able to see systemic changes after exposure to cigarette smoke. Stable serum as a biomarker calculation method has better reproducibility, sensitivity, and higher metabolite concentration than plasma. 22 Therefore, this study used serum samples in the blood.

Persistent exposure to sidestream smoke for 28 days will cause DNA damage due to uncontrolled oxidative stress in the rat body. 23 This study was conducted with groups of 4- and 8-week exposure because in that period the rats would have entered the chronic phase. 24 The exposure to cigarette smoke is done once a day using a smoking pump that exhaled sidestream fumes into rat holes with a dose of 2 cigarettes for each rat. With the same dose of cigarettes, a study by Semenzati et al showed premalignant lesions appeared after 60 days but using mainstream smoke. 25

This study used a spectrophotometer to calculate the MDA level from blood serum, which is simple, inexpensive, and widely used. In addition, spectrophotometer happens to be one of the methods available in the institution. According to Bello et al 27 and Kim et al 26 , this method provides accurate and dependable findings.

Based on the results, there was an increase in the mean value of MDA level in the T4 and T8 groups when compared with the control group. Similar to the study of Wang et al, the MDA level in the current study continued to increase even at longer times up to 24 and 36 weeks. This occurred due to the exposure to cigarette smoke, which increases ROS to high and persistent levels, upon exposing the rats with sidestream smoke. The side stream smoke will cause damage to fat membranes that are sensitive to oxidative stress, which will worsen with time. 24 27 The increase of MDA level in rats exposed to sidestream cigarette smoke occurs because ROS would cause DNA damage and reduce oxygenation in blood. 26 Nicotine in cigarette smoke can cause vasoconstriction of blood vessels because it triggers the sympathetic ganglion in the brain to produce catecholamine, which then stimulates α receptors in blood vessels. Vascular vasoconstriction is also caused by tar in blood capillaries. The occurrence of vasoconstriction in blood vessels will cause blood thickening and accumulation of ROS in blood. 28

However, when compared with a study from Kim et al, this study showed a higher increase in the MDA level, and it is assumed that this is because of the difference in the area of cigarette smoke exposure. Kim et al used one box for all treatment groups, while in this study one small box was used for one sample so that cigarette smoke will be distributed evenly to each rat. 26 Although the distribution of smoke will be homogenous, a small box of each individual rat can limit their movement and cause stressful conditions. In this situation, the rat's body will provide resistance to the stressor by activating the sympathetic nerves. The active sympathetic nerve triggers mitochondria to produce more ROS in the body along with the nicotinamide adenine dinucleotide phosphate oxidase (NOX) enzyme. The vasoconstriction of blood vessels will also trigger oxygen to become a superoxide anion in the body of the rat. The accumulation of ROS will then affect the central nervous system, especially in the central gland of the hypothalamus, pituitary gland, and adrenal gland (HPA axis), which then becomes a stressful state in the rat body. 29

The Yanxia et al study in the 4-week treatment group showed higher a MDA level compared with this study. In their study, each rat was exposed to three cigarettes per day, while this study only used two cigarettes per day. With the increasing number of cigarettes given to the rats, more smoke will contain carcinogens that expose rats and trigger an increase in the MDA level in rat blood. 30

Although there was a significant increase in the serum MDA level of Wistar rats given a longer duration of sidestream smoke exposure, another test was needed to show whether there would be a progression to malignancy. Therefore, in this study additional assessment was performed to evaluate the number of apoptotic cells in the oropharyngeal tissue of the experimental animal model given multiple-exposure duration to sidestream cigarette smoke. The decrease in the number of apoptotic cells can be a sign of an OPMD. A reduction in apoptosis in the malignant lesion is caused by DNA damage that regulates apoptosis in the process of malignant transformation. Chronic sidestream cigarette smoke also contains substances that can reduce cell apoptosis through apoptotic resistance. 8

The oropharynx was chosen as the object of this study because it is a part of the upper aerodigestive tract where the majority of HNSCC (head neck squamous carcinoma cell) cases are found. 11 12 Additionally, people exposed to sidestream cigarette smoke have a high risk of upper aerodigestive tract cancers such as oropharynx. 31 According to Viswanathan et al, the number of cells undergoing apoptosis in the oropharyngeal mucosa was determined based on observations of histological preparations under a light microscope with 400 times magnification. 15

From the results of statistical analysis, it was shown that there were significant differences between the study groups. However, these results did not support the research hypothesis, which is: the number of cells undergoing apoptosis decreased with increasing exposure time, while the result of this study showed an increase in the number of apoptotic cells with the increase in exposure time. The decrease in the number of cells undergoing apoptosis is caused by the presence of nicotine, NNK (4-methylnitrosamino)-1-(3-pyridyl)-1-butanone) in sidestream cigarette smoke, which will later bind to nAchRs (nicotine acetylcholine receptors), then stimulate phosphorylation of proapoptotic proteins (BAD, BAX) and antiapoptotic proteins (BCL2, MCL-1) through activation of several protein kinase pathways. Phosphorylation of BCL2 and MCL-1 proteins will improve its function as an antiapoptotic protein, while phosphorylation of BAD and BAX proteins will reduce its function as a proapoptotic protein. 32 33 The increase in the amount of apoptosis that occurred in this study was probably caused by an increase in the ROS level in the rat body after exposure to sidestream cigarette smoke. Excess ROS at the cellular level can cause damage to proteins, nucleic acids, lipids, membranes, and organelles, which can cause activation of the apoptotic process. 33

The immune system can also be the activator of apoptosis because the body tries to eliminate damaged cells from the body by increasing the apoptosis of damaged cells. Cytotoxic T-lymphocytes and natural-killer (NK) cells can recognize cells that have malignant transformation and cause these cells to die by apoptosis. 14 In Kesarwani et al, it was found that the number of apoptotic cells still increased at the level of mild dysplasia and moderate dysplasia. The decrease in the number of cells undergoing apoptosis occurs at the severe stage of dysplasia and carcinoma. 34 In this study, lesions in the oropharynx may be in the mild stage of dysplasia so that apoptosis remains high compared with the severe stage of dysplasia.

Variations in the molecular level between mice cause the results in each mouse sample to be different even though they are grouped in the same treatment group as the samples did not come from the same parent. This results in genetic differences that are influential in cell division such as p53 so that susceptibility to genetic mutations is different between individual rats. 35 A previous study conducted by Prasetyaningtyas et al 17 and Ayuningtyas et al 36 showed that cigarette smoke exposure can increase the dysplasia degree and induce the risk of oral cancer development due to increased number of macrophages, lymphocytes, and MMP-9 expressions in the tongue epithelium.

This study found that exposure to sidestream cigarette smoke can increase the MDA level, which can be used as a systemic biomarker, with concomitant increase in the TA count, which served as tissue alteration cellular marker. Both may be used as a marker of malignant progression because increased duration of exposure can cause an increasing number of carcinogens received by mucosal tissue so that malignant transformation of cells becomes higher. Additionally, epithelial cells responded to sidestream smoke cigarettes by increased MDA level and attempted to balance by removing cells with damaged genomes using the apoptosis mechanism. These findings are in line with the previous study by Nambiar et al 37 and Pawar et al. 38

## Conclusion

Exposure to sidestream cigarette smoke increased the TA count and MDA level of Wistar rats and may be used as a marker of malignant progression.
